# Pausing to reflect in a high-volume clinical milieu

**DOI:** 10.1007/s40037-016-0316-y

**Published:** 2016-12-22

**Authors:** Rimas V. Lukas, Dara V. F. Albert

**Affiliations:** 1 Department of Neurology, University of Chicago, Chicago, IL USA; 2 Department of Medicine, Section of Hematology & Oncology, University of Chicago, Chicago, IL USA; 3 Nationwide Children’s Hospital, Section of Pediatric Neurology, Ohio State University, Columbus, OH USA

The recent exploratory study of the role of daily reflection on the clinical learning experience by Larsen, et al. [1] and its associated editorial [2] are of significant interest. We appreciate the authors’ emphasis on the importance of finding time to pause at the end of busy days of clinical education and clinical care and the associated benefits of this on the building of clinical knowledge and skill. One could suspect that these benefits would not only apply to the third year medical students participating in a paediatric neurology clerkship but to other trainees as well as faculty physicians. This of course needs to be balanced against the large volume of clinical material which trainees need to master as well as the substantial number of patient care responsibilities which both trainees and faculty need to address on a daily basis. It also needs to take into account the favourable aspects of participating in the care of a large volume of patients [3]. Evaluating a substantial volume of patients provides the trainee with a broad clinical experience. Seeing a number of patients with the same disease exposes the trainee to the nuances of the disease process within the context of human beings and provides the repetition which the authors note is important for the educational experience. A high volume clinical learning experience and time routinely allotted for reflection are not mutually exclusive. The pairing of these two elements has the potential to further augment the learning experience by consolidating the day’s knowledge, thematically tying together distinct clinical experiences, and providing an opportunity to trouble-shoot means of avoiding the day’s mistakes in the future. We look forward to future explorations of the questions posed by this exploratory study.
